# Higher Risk Myelodysplastic Syndromes in Patients with Well-Controlled HIV Infection: Clinical Features, Treatment, and Outcome

**DOI:** 10.1155/2016/8502641

**Published:** 2016-01-20

**Authors:** Bradley T. Williamson, Heather A. Leitch

**Affiliations:** ^1^Medicine, St. Paul's Hospital and the University of British Columbia, Vancouver, BC, Canada V6Z 2A5; ^2^Hematology, St. Paul's Hospital and the University of British Columbia, Vancouver, BC, Canada V6Z 2A5

## Abstract

*Introduction*. In advanced HIV prior to combination antiretroviral therapy (ART), dysplastic marrow changes occurred and resolved with ART. Few reports of myelodysplastic syndromes (MDS) in well-controlled HIV exist and management is undefined.* Methods*. Patients with well-controlled HIV and higher risk MDS were identified; characteristics, treatment, and outcomes were reviewed.* Results*. Of 292 MDS patients since 1996, 1 (0.3%) was HIV-positive. A 56-year-old woman presented with cytopenias. CD4 was 1310 cells/mL and HIV viral load <40 copies/mL. Bone marrow biopsy showed RCMD and karyotype included del(5q) and del(7q); IPSS was intermediate-2 risk. She received azacitidine at 75% dose. Cycle 2, at full dose, was complicated by marrow aplasia and possible AML; she elected palliation. Three additional HIV patients with higher risk MDS, aged 56–64, were identified from the literature. All had deletions involving chromosomes 5 and 7. MDS treatment of 2 was not reported and one received palliation; all died of AML.* Conclusion*. Four higher risk MDS in well-controlled HIV were below the median age of diagnosis for HIV-negative patients; all had adverse karyotype. This is the first report of an HIV patient receiving MDS treatment with azacitidine. Cytopenias were profound and dosing in HIV patients should be considered with caution.

## 1. Introduction

The myelodysplastic syndromes (MDS) are clonal hematopoietic stem cell disorders characterized by ineffective hematopoiesis and risk of progression to acute myeloid leukemia (AML) [1, 2]. The diagnosis of MDS is made according to World Health Organization (WHO) [3] criteria and prognosis determined by the International Prognostic Scoring System (IPSS) [4] and other scores. MDS is thought to result from mutations in the pluripotent stem cell [5, 6], resulting in dysplasia and ineffective hematopoiesis. Mutations in genes coding the RNA splicing machinery [7], haploinsufficiency of ribosomal proteins [8], telomere disruption [9], abnormal microRNA expression [10], aberrant DNA methylation patterns [11], and immune dysregulation [12, 13] may play a role in MDS pathogenesis.

The relationship between MDS and the immune system is poorly understood; however, cytokine dysregulation [14, 15] and impaired cellular immunity [16, 17] have been implicated in MDS initiation, development, and progression. Autoimmune manifestations (AIM) may occur in up to 30% of MDS patients [18, 19] and may be associated with MDS onset at a younger age and adverse cytogenetics [19–21]. Patients with immune disorders and/or receiving immunosuppressive medications are more likely to be diagnosed with MDS or AML [22, 23], and paraneoplastic inflammatory syndromes may occur concomitant with MDS diagnosis [20, 24–27].

The protease inhibitors became available for the treatment of HIV infection in 1996, introducing the era of combination antiretroviral therapy (ART). For patients with advanced HIV infection in the pre-ART era, dysplastic marrow changes were a frequent finding and these resolved with antiretroviral treatment [28, 29]. There are few reports of MDS in well-controlled HIV infection [30–32] and an approach to management is largely undefined. Here, we describe the characteristics and clinical course of a patient presenting with higher IPSS risk MDS in the setting of well-controlled HIV infection and compare to the features of three patients reported in the literature. We discuss the effect of immune dysregulation on the development of MDS, the possible role of HIV and/or ART in MDS development, and treatment of our patient with azacitidine.

## 2. Methods

Patients with MDS in well-controlled HIV infection were identified from the MDS clinical database and the literature and reviewed for MDS and HIV clinical and laboratory characteristics, treatment, and outcome.

This review was conducted in accordance with the requirements of the Institutional Research Ethics Board.

## 3. Results

A 56-year-old woman with a longstanding history of HIV infection, well-controlled on ART, was referred with fatigue and cytopenias. Eastern Cooperative Oncology Group (ECOG) performance status (PS) was 1 [33]. On atazanavir, emtricitabine, and tenofovir, the CD4 count was 1310 cells/mL with a CD4 fraction of 29% and HIV viral load (VL) was undetectable at <40 copies/mL. Sequential complete blood count with differential revealed a white blood cell (WBC) count of 11.3 × 10^9^/L, neutrophils fluctuating between 0.18 × 10^9^/L and 1.9 × 10^9^/L, hemoglobin (Hb) of 75 g/L, and platelet count (PLTS) of 132 × 10^9^/L. Morphology review revealed giant platelet forms and pelgeroid changes. A bone marrow aspirate and biopsy (BMBx) showed a hypercellular marrow (100% cellularity) with trilineage dysplasia, 4% blasts, and cytogenetic analysis revealed a complex karyotype including deletion of 5q and 7q, a dicentric chromosome (9; 12) with break points at 9q and 12p, a marker chromosome, a ring chromosome, and two tetraploid metaphases. A diagnosis of refractory cytopenia with multilineage dysplasia (RCMD) was made and the IPSS score was intermediate-2 risk.

While awaiting cytogenetic analysis to determine IPSS risk, she presented with high fevers, drenching sweats, rigors, and chills. A chest X-ray showed pulmonary infiltrates. ECOG PS was 3. She was admitted to hospital and an extensive workup including bronchoscopy and repeat BMBx revealed no infectious cause and no sign of MDS progression. The symptoms resolved spontaneously over the following four weeks.

**Figure 1:**
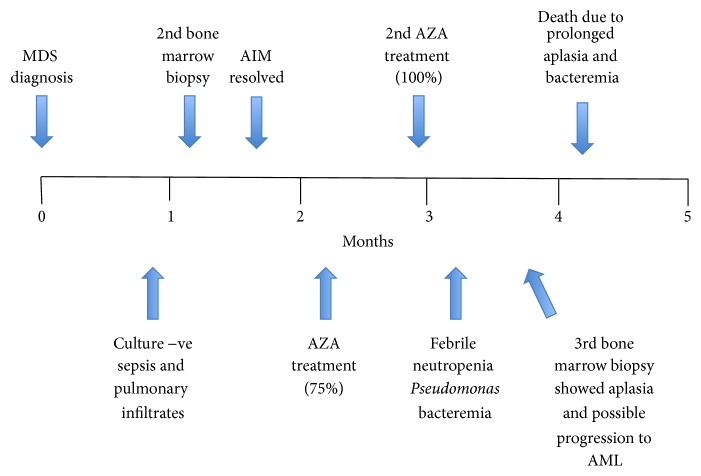
Clinical course of a patient presenting with higher IPSS risk MDS in the context of well-controlled HIV infection. AIM: autoimmune manifestations; AML: acute myeloid leukemia; AZA: azacitidine; IPSS: International Prognostic Scoring System; MDS: myelodysplastic syndrome.

She received transfusion support and two cycles of azacitidine, with a plan to bridge to allogeneic hematopoietic stem cell transplantation; the antiretroviral regimen was continued unchanged throughout. The first azacitidine cycle was given at a dose reduction of 25% due to uncertainty as to how it would be tolerated in the setting of HIV and ART. Throughout this cycle, the patient was clinically very well with an ECOG PS of 0. The nadir counts of five intervening measurements were neutrophils 0.1, hemoglobin 75, and platelets 13. She received transfusion of 2 units of packed red blood cells and one pack of platelets. The second cycle of azacitidine was given at 100% dose and was complicated by prolonged and profound cytopenias lasting over four weeks (neutrophils 0.1 × 10^9^/L, transfused Hb 83 g/L, and transfused PLTS 2 × 10^9^/L) and* Pseudomonas* bacteremia.* Aspergillus* infection and atypical mycobacterial infection were suspected but not proven. A third BMBx showed aplasia and possible progression to acute erythroleukemia. The patient elected comfort care and died. See Figure 1 for a summary of the patient's course.

There are three other cases in the literature of patients with reasonable HIV control on ART (CD4 > 200 cells/mL with negative HIV VL, *n* = 2; and CD4 > 500 with VL uncertain, *n* = 1) presenting with MDS that is clearly reported to be higher IPSS risk (see Table 1) [30, 32]. At ages 56, 60, and 63, like our patient, all were below the median age of MDS diagnosis in the HIV-negative population. All four patients had a complex karyotype including deletions involving chromosomes 5 and 7. The marrow blast count was not reported in the first 2 patients and was 12% in the third. Treatment for MDS received by two patients was not reported and the third received supportive care; all died of AML progression within three to six months of MDS diagnosis.

## 4. Discussion

Dysplastic changes to the marrow were a common finding in advanced HIV infection prior to the advent of protease inhibitors and ART [28, 29], and these generally resolved with the institution of effective HIV treatment. Our patient with higher risk MDS had a longstanding history of HIV infection which was well controlled with ART. Other reports have recently emerged [30–32] describing patients presenting with MDS in well-controlled HIV, suggesting that HIV-positive patients are now living long enough to develop age-related conditions such as MDS.

Several reports have documented an association between AIM and MDS [20, 24–27]. Acute systemic vasculitis may present as culture negative fever and pulmonary infiltrates, which our patient exhibited, and an autoimmune cause was suspected though not conclusively demonstrated [20, 34]. Some inflammatory syndromes may precede the diagnosis of MDS [23], suggesting a role in MDS development and progression and raising the possibility that residual immune dysregulation in treated HIV may contribute to MDS development. Immune dysregulation in MDS includes abnormal levels and activity of tumor necrosis factor alpha (TNF-*α*), transforming growth factor beta (TGF-*β*) [35], interferon- (IFN-) *γ*, and interleukin- (IL-) 6 [14]. TNF-*α* facilitates apoptosis of CD34-positive cells [36] and results in upregulation of proinflammatory cytokines [37], leading to decreased B-cell proliferation and natural killer (NK) cell dysfunction. Deletions and mutations in the tumor suppressor interferon regulatory factor-1 (IRF-1) gene, as well as alternative splicing of IRF-1 mRNA, have been found in MDS [38, 39]. Aberrant activity of the immune regulatory *γδ* T cells (CD8+) has been shown [36, 40]. In higher risk MDS, NK cell levels are decreased, and regulatory T cells (also CD8+) are increased [41, 42], both presumably leading to aberrant immune surveillance and MDS progression [43–46]. Thus, many levels of immune dysregulation may contribute to the development of MDS. It is possible that some or all of this immune dysregulation may differ in extent or frequency in HIV-positive patients. Though information on cytokine levels in ART treated patients is incomplete and sometimes conflicting, TNF-*α*, IFN-*γ*, IL-6, and T regulatory cells have been reported to be increased [47–50] and one study shows an association between increased CD8 cell numbers and decreased NK cell function [51]. While our patient's CD4 count was well preserved at 1,310 cells/mL, the CD4 fraction was only 29%, indicating a CD8 count of 4517 cells/mL. We do not have information on this patient's CD8 subtypes but speculate that her high CD8 count may have allowed AIM, and possibly MDS, development. There are similar reports of an association between AIM in MDS and infection with the HIV-related retrovirus human T lymphotropic virus-1 (HTLV-1) [52].

The prognostic impact of the presence of AIM in MDS is unclear [20, 21]. Immunosuppressive medications such as prednisone, cyclophosphamide, and azathioprine have been used [20, 53–55]; however, remissions are not sustained. Paraneoplastic AIM may respond to azacitidine, which may have immunomodulatory effects such as reduction of immune-mediated cytotoxicity and increased IFN-gamma production [25, 56].

Our patient and the three other patients with higher IPSS risk MDS and well-controlled HIV infection reported in the literature all had a complex karyotype, and all had deletions involving both chromosomes 5 and 7. The significance of this finding is uncertain; however, both abnormalities of these particular chromosomes and complex cytogenetic abnormalities are common in therapy-related MDS [57]. Some data suggest that protease inhibitors may decrease cellular efflux of toxins, and it is possible that this may contribute to the development of MDS in general and specific chromosomal abnormalities in particular [58]. Similarly, while azacitidine is not metabolized by the hepatic cytochrome P450 system and would not be expected to interact directly with antiretroviral medications, it is possible that increased cytotoxicity could result from decreased cellular efflux of this agent. Equally possible, however, is that marrow suppression from ART may be additive to marrow suppression from azacitidine; one study in NHL showed more cytopenias with chemotherapy in conjunction with PI-based ART compared with PI-sparing regimens [59].

Reports from the pre-ART era indicate direct infection of hematopoietic progenitor cells and bone marrow stromal cells with HIV [60, 61], which could contribute to cytopenias by decreasing the cellularity of the marrow, though whether these phenomena occur in well-controlled HIV infection is unclear. Our patient's marrow was hypercellular, as was the marrow of case 3; the marrow cellularity of cases 1 and 2 was not reported. Of particular interest in this setting are reports of HIV inhibitory activity of azacitidine and related compounds in preclinical models [62–64]. It may prove in future that ARV regimens may be modified in patients receiving azacitidine or related compounds for MDS in HIV infection, though this approach should be considered investigational. Patients with well-controlled HIV and other hematological malignancies such as non-Hodgkin lymphoma (NHL) can currently be expected to have treatment outcomes equivalent to the HIV-negative population [65–68]. However, in HIV NHL patients, bone marrow function may be more borderline than in HIV-negative patients, for example, requiring more frequent use of cytokine support with chemotherapy [65]. Our patient was treated with azacitidine with the intent to induce remission and/or delay AML progression and bridge to allogeneic hematopoietic stem cell transplantation. While she tolerated the first cycle of azacitidine, given at a 25% dose reduction, well, the second cycle resulted in prolonged aplasia and serious infections. Guidelines suggest that dose reductions of azacitidine should be avoided if possible [69]; however, in HIV-positive patients with MDS, consideration of dose reductions may be prudent.

Finally, there are several reports of patients with MDS in HIV infection, including lower risk patients, who progressed rapidly (within 4–14 months) to AML following MDS diagnosis [30, 70, 71]. This suggests that the IPSS may, at least in some HIV-positive patients, underestimate the aggressiveness of the MDS and that these patients may be appropriate for vigilant monitoring and/or early intervention.

## 5. Conclusion

Of 292 MDS patients at our center in the ART era, one presented with higher IPSS risk MDS in well-controlled HIV infection, which may indicate that HIV-positive patients are currently living long enough to develop age-related complications such as MDS. This and three other reported patients with higher risk MDS in HIV were younger than HIV-negative MDS patients, and all patients had a complex karyotype and deletions involving chromosomes 5 and 7; this finding is of uncertain significance. To our knowledge, this is the first report of an HIV-positive patient receiving specific treatment for higher risk MDS with azacitidine. The patient died of sepsis with prolonged aplasia following azacitidine at 100% dose, which may indicate that, like HIV patients receiving ART and chemotherapy for lymphoma, cytopenias may be more profound than in HIV-negative patients, and dosing should be considered with caution. Our patient developed culture negative sepsis prior to MDS treatment, in keeping with reports of AIM leading to or presenting as a paraneoplastic manifestation of MDS. Whether AIM are more frequent in HIV-positive patients due to cellular and cytokine dysregulation is an unanswered question. MDS should remain on the differential diagnosis of patients with well-controlled HIV infection and cytopenias. These patients should be considered for the usual MDS therapies, though dose reductions with careful dose escalation as tolerated, should be considered.

## Figures and Tables

**Table 1:** Clinical features, treatment, and outcome of 4 patients with higher IPSS risk MDS in the setting of well-controlled HIV infection.

Case	Age (years)	PMHx	ART	CD4 (cells/mL)	CD4%	HIV VL (copies/mL)	FAB or WHO MDS diagnosis	Marrow blast count (%)	Cytogenetic analysis	IPSS score	MDS treatment	Follow-up (months)	Outcome
1^1^	56	Hepatitis C	Abacavir Efavirenz Lamivudine	206	NR	Neg	RAEB^2^	NR	Complex including del(5q) and -7	≥int2	NR	4	Progressed to AML

2^1^	60	NHL	Abacavir Lamivudine Lopinavir	500	NR	NR	RAEB^2^	NR	Complex including del(5q) and -7	≥int2	NR	6	Progressed to AML

3^3^	63	None	Tenofovir Efavirenz Emtricitabine	296		<40	RAEB-2^4^	12	Complex including -5 and -7	High	None	3	Progressed to AML

4	56	None	Atazanavir Emtricitabine Tenofovir	1310	29	<40	RCMD^4^	4	Complex including del(5q) and del(7q)	int2	AZA (2 cycles)	4	Septic death with possible AML progression

AML: acute myelogenous leukemia; ART: antiretroviral therapy; AZA: 5-azacitidine; del: deletion; FAB: French-American-British; HIV: human immunodeficiency virus; int: intermediate; IPSS: International Prognostic Scoring System; MDS: myelodysplastic syndrome; neg: negative; NHL: non-Hodgkin lymphoma; NR: not reported; PMHx: past medical history; WHO: World Health Organization; VL: viral load; ^1^[30];  ^2^FAB; ^3^[32];  ^4^WHO.
